# Effect of planned school breaks on student absenteeism due to influenza‐like illness in school aged children—Oregon School District, Wisconsin September 2014–June 2019

**DOI:** 10.1111/irv.13244

**Published:** 2024-01-16

**Authors:** Cecilia He, Derek Norton, Jonathan L. Temte, Shari Barlow, Maureen Goss, Emily Temte, Cristalyne Bell, Guanhua Chen, Amra Uzicanin

**Affiliations:** ^1^ University of Wisconsin Madison Wisconsin USA; ^2^ Centers for Disease Control and Prevention Atlanta Georgia USA

**Keywords:** influenza, K‐12, respiratory infection, school breaks, student absenteeism, viral surveillance

## Abstract

**Background:**

School‐aged children and school reopening dates have important roles in community influenza transmission. Although many studies evaluated the impact of reactive closures during seasonal and pandemic influenza outbreaks on medically attended influenza in surrounding communities, few assess the impact of planned breaks (i.e., school holidays) that coincide with influenza seasons, while accounting for differences in seasonal peak timing. Here, we analyze the effects of winter and spring breaks on influenza risk in school‐aged children, measured by student absenteeism due to influenza‐like illness (a‐ILI).

**Methods:**

We compared a‐ILI counts in the 2‐week periods before and after each winter and spring break over five consecutive years in a single school district. We introduced a “pseudo‐break” of 9 days' duration between winter and spring break each year when school was still in session to serve as a control. The same analysis was applied to each pseudo‐break to support any findings of true impact.

**Results:**

We found strong associations between winter and spring breaks and a reduction in influenza risk, with a nearly 50% reduction in a‐ILI counts post‐break compared with the period before break, and the greatest impact when break coincided with increased local influenza activity while accounting for possible temporal and community risk confounders.

**Conclusions:**

These findings suggest that brief breaks of in‐person schooling, such as planned breaks lasting 9–16 calendar days, can effectively reduce influenza in schools and community spread. Additional analyses investigating the impact of well‐timed shorter breaks on a‐ILI may determine an optimal duration for brief school closures to effectively suppress community transmission of influenza.

## 1 INTRODUCTION

School‐aged children are often recognized as primary drivers of influenza transmission within communities,^1^ and in the fall of 2009 school reopening dates were associated with the local surges of pandemic influenza.^2^ Children frequently have larger social networks,^3^, ^4^ experience prolonged viral shedding,^5^ have lower coverage rates for influenza vaccine,^6^ and may lack sufficient preexisting immunity for herd effects.^7^ Although most of the frequent influenza infections among school‐aged children are mild to moderate, some children can still develop serious influenza‐related complications following infection.^8^ During the 2017–2018 influenza season, there were an estimated 11.5 million cases of influenza in children and over 48,000 pediatric hospitalizations in the United States alone.^8^

The rapid evolution and wide variability of the influenza virus contribute to the challenges of control. Normal efforts in disease prevention, such as vaccination, are hampered as vaccines must be updated and administered annually to account for changes in circulating viruses,^9^ leading to varying levels of effectiveness from year to year.^10^ Thus, it is important to consider alternative strategies to control outbreaks, especially during seasons when vaccine effectiveness is suboptimal, or when a well‐matched vaccine is not yet available (e.g., in the early stages of a pandemic).

School closures include planned breaks in instruction for holidays or teacher training, and unscheduled breaks due to weather, safety, or other emergencies. With regard to their anticipated effects on influenza transmission, school closures are considered a nonpharmaceutical intervention (NPI) only when implemented sufficiently early relative to the start of an outbreak (i.e., before influenza becomes widespread in schools and surrounding communities).^11^ Effectiveness of preemptive school closures has been extensively studied and scrutinized in systematic literature reviews.^12^, ^13^ In contrast, reactive school closures—implemented only after influenza is widespread in schools—are not considered NPI, but rather a consequence of the disease^11^ because epidemiologic studies have not found them to effectively reduce medically attended influenza (MAI) in surrounding communities.^14^, ^15^, ^16^ Studies noted reactive closures to have no statistically significant impact on overall influenza‐like illnesses (ILIs) rates.^14^, ^15^ In fact, these unplanned closures often have socioeconomic consequences and may further introduce challenges to households, such as making alternative childcare arrangements and loss of access to school lunch programs.^16^

**FIGURE 1:**
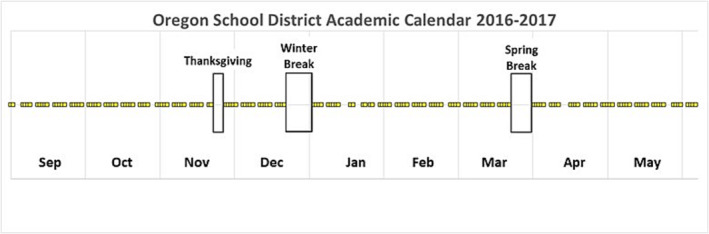
Representative kindergarten through 12th grade academic year in the Oregon School District (OSD: Dane County, WI) showing school days (yellow squares) and non‐school days for the 2016–2017 academic year. Longer‐duration planned school breaks for Thanksgiving, winter break, and spring break are demonstrated by empty boxes.

Schools close for regularly scheduled or planned breaks (holidays) throughout the academic year (Figure 1). At least one earlier study reported that such planned school breaks may interrupt the dynamics of seasonal influenza by changing social contact patterns among children.^17^ Such closures have been associated with a reduction in the reproduction number relative to when school is in session, leading to reduced transmission.^18^, ^19^ However, the precise impact of these breaks on seasonal influenza remains unclear. Few studies have investigated the effect of planned school closures on local transmission, and no studies currently assess the impact of numerous breaks within an academic year to account for potential seasonal differences in the timing of circulation.

From our causal observations on reduced influenza‐like illness absenteeism (a‐ILI) following scheduled school break periods and unscheduled weather‐related closures (e.g., snow days), we hypothesized that longer periods, such as winter and spring break, may substantially reduce absenteeism. Our goal, therefore, was to evaluate the effects of scheduled breaks in the academic calendar as a potential intervention to reduce a‐ILI due to ILI. Such a finding would set the stage for potential alterations in school schedules and an anticipatory (proactive) versus reactive approach, with the benefit of not losing scheduled school days. To account for multiple breaks and seasonal timing, we investigated the role of regularly scheduled school breaks on ILI within a single school district over the course of five academic years. We assessed rates of a‐ILI during 2‐week periods leading up to and following scheduled winter and spring breaks.

## 2 METHODS

### 2.1 Orchards

The ORegon CHild Absenteeism due to Respiratory Disease Study (ORCHARDS) is a prospective, observational study of kindergarten through 12th grade (K‐12) student absenteeism and influenza in the Oregon School District (OSD), Dane County, located in southcentral Wisconsin. The primary goal of ORCHARDS is to develop a system to monitor cause‐specific, K‐12 absenteeism on a daily basis and to assess its usability for early detection of influenza and ILI transmission in schools and in the community. The overall methodology of ORCHARDS is detailed elsewhere.^20^

### 2.2 Population

The OSD comprises six public schools with an enrollment estimated at 4091 students (18% of the total population) during the 2018–2019 school year.^21^ The district's overall population is estimated at 23,000 and is less racially and ethnically diverse, wealthier, and more educated than the average community in the United States.^22^

### 2.3 Data collection

Parents/guardians are required to report absences using an automated telephone system and are prompted to report respiratory symptoms. The OSD records absenteeism in Infinite Campus® (https://www.infinitecampus.com), a commercially available electronic student information system. For the purpose of this study, in 2014 the OSD Information Technology staff added an option within the system that allowed entry of student absenteeism characterized as a‐ILI. We defined absence as missing any part of the school day. We defined ILI as the presence of fever and at least one of the following symptoms: cough, sore throat, nasal congestion, or runny nose^20^ as reported by a parent/guardian on the telephone system. The daily count of a‐ILI was the primary outcome measure for this study. The annual number of a‐ILI days has been stable (1141—1609) over the study period.^20^

### 2.4 Data extraction

The OSD developed an automated process to extract daily counts of student absences by school, grade, and type of absence. Data were sent on a daily basis to ORCHARDS researchers using a secure file transfer (ftp) site. No personally identifiable information was included, and the data were fully compliant with the Family Educational Rights and Privacy Act (FERPA 20 U.S.C. § 1232g; 34 CFR Part 99).

### 2.5 Community risk

The Wisconsin component of the Influenza Incidence Surveillance Project (W‐IISP) is a long‐standing, independent influenza surveillance system that assesses MAI in and around OSD.^23^ The system has been in continuous operation since October 2009 and is organized by the ORCHARDS research team. W‐IISP includes five primary care clinics, one of which is located in the OSD and four that are located in communities surrounding OSD. The clinics conduct active laboratory‐supported surveillance for influenza and other respiratory viruses in patients presenting with acute respiratory illnesses. Weekly counts of laboratory‐confirmed MAI served as a proxy for underlying community influenza risk in this analysis.

### 2.6 Timing of regularly scheduled major school breaks

The winter holiday (including Christmas and New Year's Day) at the OSD is relatively fixed in time, occurring in late December and early January, extending between 10 and 16 days, including weekend days (Table 1). The timing of spring break is more variable depending upon the year but is fixed in length at 9 days (including weekend days).

**TABLE 1:** Length (in days) of winter, spring, and pseudo‐breaks from the 2014/2015 to 2018/2019 academic years. Specific dates of each break are provided in parentheses.

Year	Winter break	Spring break	Pseudo‐break
2014/2015	16 (12/20/2014–1/04/2015)	9 (3/28/2015–4/05/2015)	9 (2/21/2015–3/01/2015)
2015/2016	12 (12/23/2015–1/03/2016)	9 (3/19/2016–3/27/2016)	9 (2/13/2016–2/21/2016)
2016/2017	11 (12/23/2016–1/02/2017)	9 (3/25/2017–4/02/2017)	9 (2/18/2017–2/26/2017)
2017/2018	10 (12/23/2017–1/01/2018)	9 (3/24/2018–4/01/2018)	9 (2/17/2018–2/25/2018)
2018/2019	11 (12/22/2018–1/01/2019)	9 (3/23/2019–3/31/2019)	9 (2/16/2019–2/24/2019)

### 2.7 Pseudo‐breaks as a control

**FIGURE 2:**
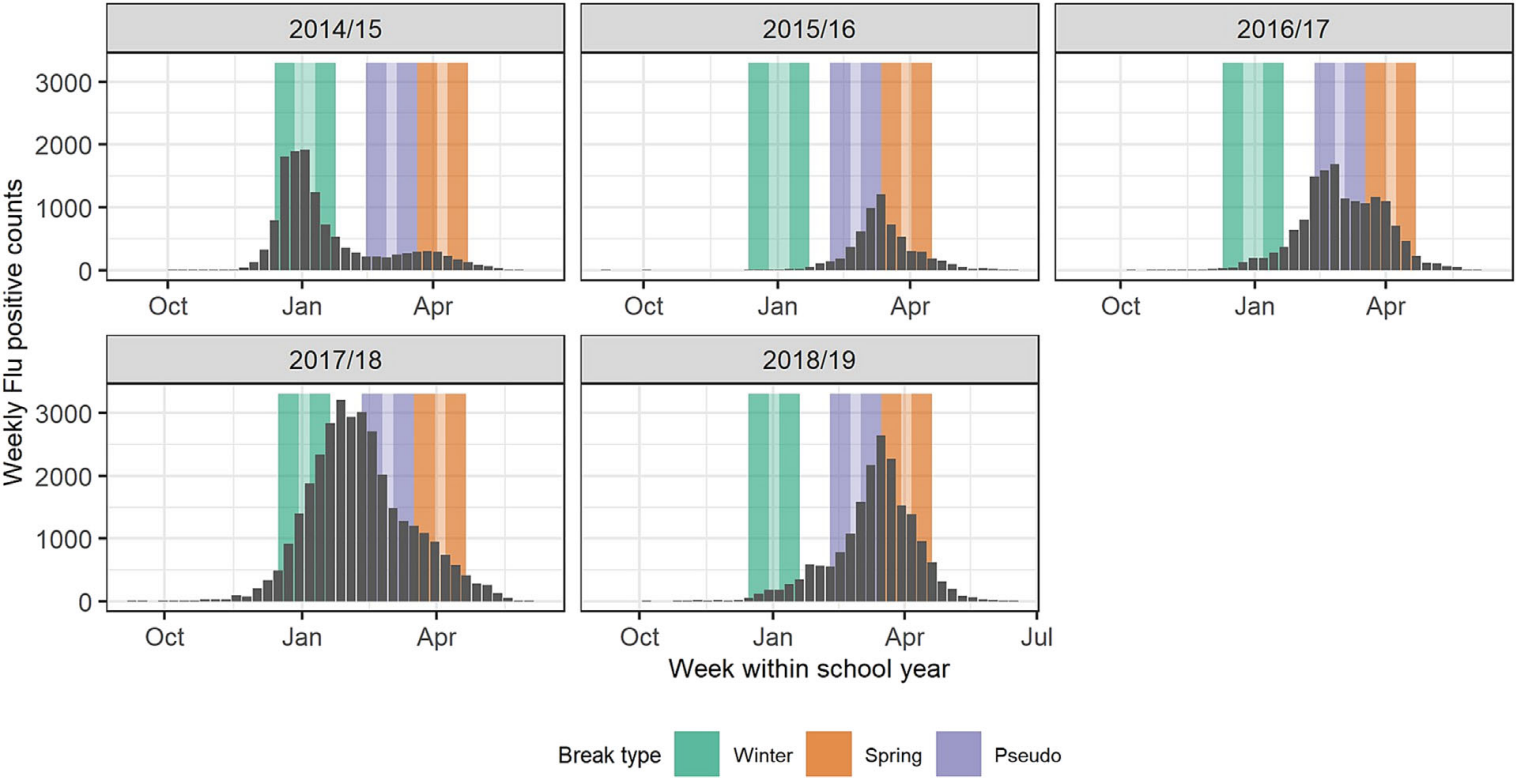
Weekly counts of Wisconsin influenza A and influenza B detections combined for the five academic years in this study. Influenza surveillance data were provided by the Wisconsin State Laboratory of Hygiene. Lightly shaded vertical bands demonstrate the actual timing of winter breaks (green) and spring breaks (orange) for each academic year/influenza season in this study. Nine‐day pseudo‐breaks between winter and spring breaks each year were introduced in this analysis to support any findings of impact from the planned breaks; they are shown as purple bars. Dark shaded bands demonstrate the 2‐week assessment periods before and after winter breaks (green), spring breaks (orange), and pseudo‐breaks (purple).

We introduced into the analysis “pseudo‐breaks” of 9 days' duration between winter and spring break each year and starting 5 weeks before the spring break, when school was actually in session, to support any findings of the true impact of the planned breaks. The timing of regularly scheduled winter and spring breaks, along with pseudo‐breaks, is presented in Figure 2 against the backdrop of statewide laboratory‐confirmed influenza detections. This pseudo‐break can be thought of as similar to a control group; the purpose is to act as a point of comparison to the actual winter and spring break associations. If the “effect” of a pseudo‐break between the two real brakes is different in significance and/or direction than the effects of real breaks under the same analysis setup, then this lends greater confidence to any findings on the real breaks.

### 2.8 Statistical analysis

Analyses were performed on absentee data from five consecutive academic school years (September 2, 2014–June 12, 2019). The primary outcome measure was the number of a‐ILI days in the 2 weeks before and after the regularly scheduled school break. Absenteeism due to ILI has been validated as an acceptable marker for influenza through the home visit component in ORCHARDS.^20^

The Cochran–Mantel–Haenszel (CMH) test was used to measure the crude association between a‐ILI and the 2‐week period before versus after each break period, stratified by school year, to help appropriately account for steadily increasing enrollment sizes over time. Exposure was defined as whether the a‐ILI count occurred during the 2‐week period before a break (not exposed) or after a break (exposed). Cases were defined as the sum of a‐ILI counts for each pairing of the school year and the 2‐week time periods before versus after a break. The number of controls (not absent due to ILI) in each of these 2‐week periods was defined as the number of students enrolled in OSD for that school year, multiplied by the number of school days in attendance during those 2‐week periods, minus the number of a‐ILI cases in that same period. Results from these analyses are on the odds ratio scale for a‐ILI risk, with the after‐break odds in the numerator.

Generalized linear regression models (GLM) were used to assess the relationship between a‐ILI counts and before‐ versus after‐break periods while accounting for the community's underlying influenza risk. A Poisson distribution was assumed with the outcome of daily a‐ILI counts and the canonical natural log link function used. GLM model results and effects are interpreted on the scale of proportional change (PC) and are interpreted in the same fashion as the incident rate ratio (IRR). The natural log of the OSD enrollment number for that school year was used as an offset to account for varying enrollment numbers. Time from break was accounted for within these 2‐week periods, measured in days. For the period before a break, days were counted going backwards in time from the first date of the break. For the period after a break, days were counted going forward in time from the last date of the break (i.e., break periods are “Day 0,” school days before a break are negative days, and school days after a break are positive days). Covariates used in this model included community‐level influenza risk, the linear effect of time, the quadratic effect of time, an indicator of before or after break, an interaction between the break indicator and linear time effect, and an interaction between the break indicator and the quadratic time effect. The use of quadratic time in the model was based on a graphical examination of the bivariate log(a‐ILI) versus time trend in these data, and having some concern for quadratic trends being present. As such, school breaks might be affecting not only the a‐ILI risk level (school break main effect) but also the linear and quadratic time trends (interactions of school breaks with these time components).

The community‐level influenza risk was represented as a weekly measure of influenza risk in the community. This was calculated by summing the number of MAI instances in the community data set for the first 7‐day period (Week 1) before and after break and the second 7‐day period (Week 2) before and after break for a total of 20 such calculations at the community level (4 weeks calculated for each of the five school years analyzed), for each break type (winter and spring).

The analysis approach above for winter and spring breaks was also applied to the pseudo‐break period that was introduced 5 weeks prior to each spring break when school remained in session. This pseudo‐break serves as a control to assess for time as a potential confounder. The estimates predicted by the model for the pseudo‐break were compared with the results from the winter and spring break analyses to help assess the true impact of school breaks on a‐ILI. Unadjusted versions of the models with only the before/after break main effect as a covariate (still including yearly enrollment as an offset) were examined, and results were included in the supplement.

Regression model diagnostics did not reveal any major concerns for heteroskedasticity, trends in the residuals, or non‐normally distributed residuals. Overdispersion was a concern in all of the regression models, with dispersion parameter estimates greater than the assumption of 1 for Poisson's GLM; all model standard errors and inferences were conducted using the estimated parameter from the fitted model to account for this overdispersion.

## 3 RESULTS

### 3.1 Absenteeism due to ILI

**FIGURE 3:**
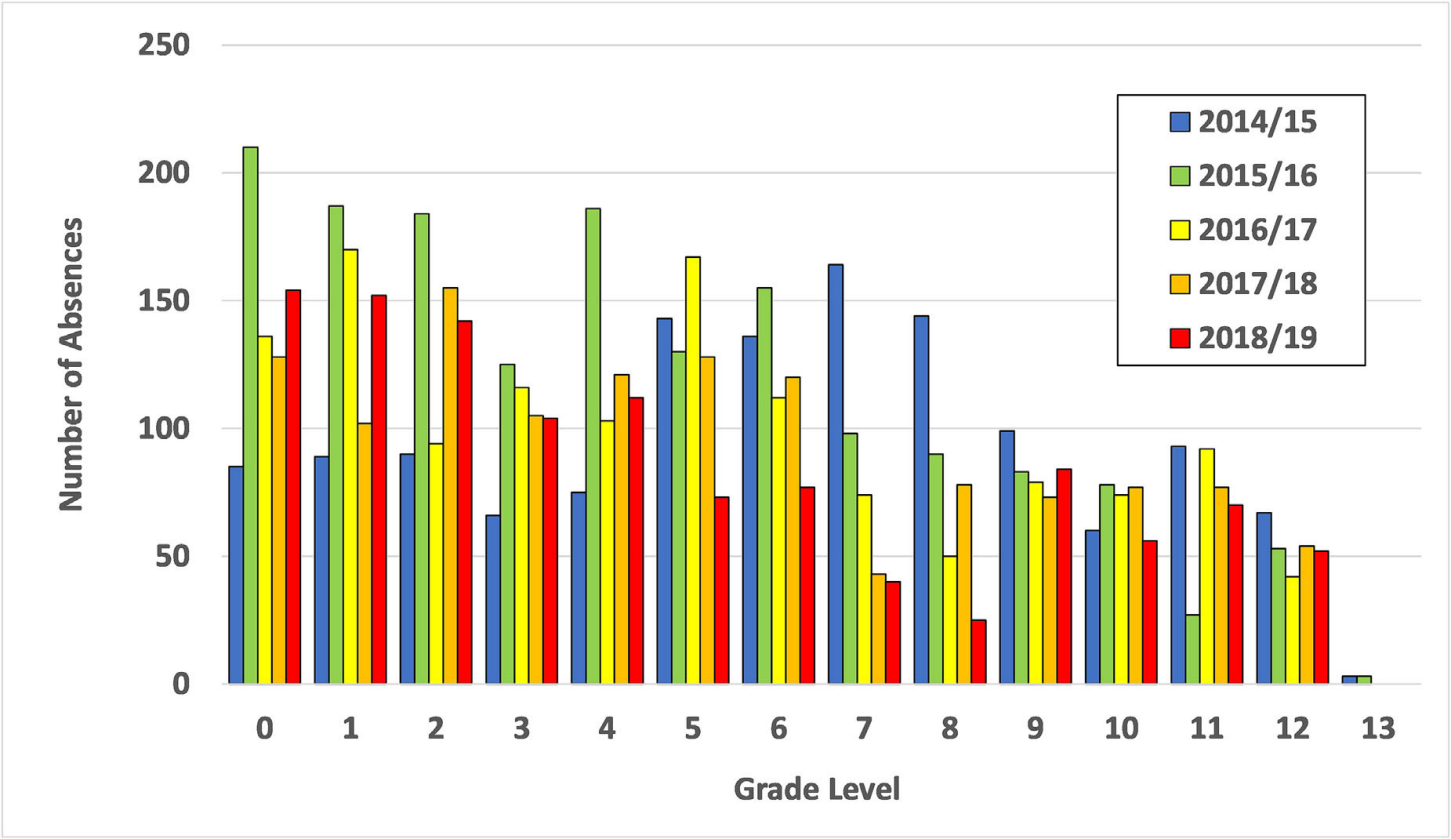
Distribution of number of absences due to influenza‐like illness (a‐ILI) per study year across all grade levels in the Oregon School District. 0 = *kindergarten*. 13 = *students eligible to remain in the public school system past age 18 years*.

Across the five academic years, the mean tallies of a‐ILI for the 2‐week periods before winter and spring breaks were 130.4 (range 51–262) and 151.4 (range 69–275), respectively. The mean a‐ILI tallies for the periods after winter and spring breaks were 82 (range 33–152) and 49.6 (range 33–74), respectively. Comparatively, the 2 weeks before pseudo‐breaks had an a‐ILI average of 106 (range 43–200), and the 2 weeks after pseudo‐breaks had a‐ILI average of 100.8 (range 71–131). The grade distributions of a‐ILI are displayed in Figure 3, showing higher levels of a‐ILI reported among students in 4K and elementary schools, in comparison to middle and high schools.

### 3.2 Crude association between school breaks and a‐ILI

**FIGURE 4:**
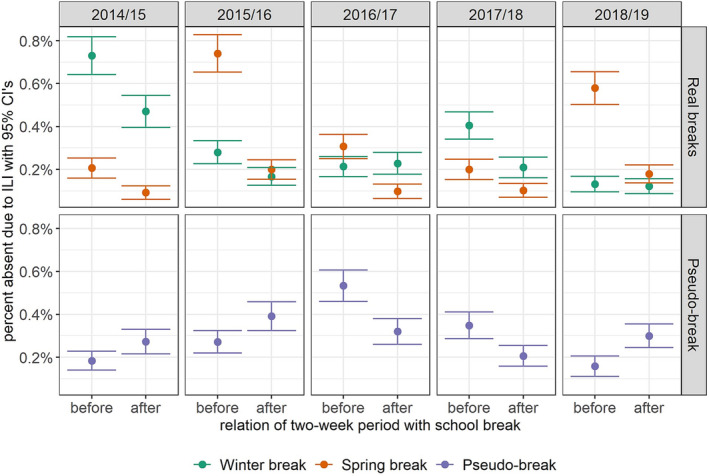
Proportion of students absent due to influenza‐like illness in the 2‐week periods before and after winter breaks (green—upper panel), spring breaks (orange—upper panel), and pseudo‐breaks (purple—lower panel) in each of five academic years. Nine‐day‐long pseudo‐breaks between winter and spring breaks each year were included in this analysis to act as control periods for comparison. The 95% confidence intervals are demonstrated by brackets.

The 2‐week after‐break period was associated with a statistically significant decrease in the odds of a‐ILI compared with the 2‐week before‐break period. The CMH test estimated an odds ratio of 0.679 (95% CI: 0.600–0.769; *p* < 0.001) following winter breaks and 0.327 (95% CI: 0.283–0.378; *p* < 0.001) following spring breaks. The crude a‐ILI counts for each school year, occurring before versus after breaks, are depicted in Table 2. Differences in a‐ILI proportions in the 2 weeks before and after each true break varied every school year (Figure 4). While several of the yearly school breaks had a clear difference in the a‐ILI proportions, not every yearly break displayed a difference.

**TABLE 2:** Absenteeism counts before and after the winter, spring, and pseudo‐breaks over five academic years for influenza‐like illness (ILI)‐associated absenteeism (a‐ILI) and the complement of this number (all other students in attendance or absent for other reasons). Odds ratios (OR) comparing after break to before break counts calculated using the Mantel–Haenszel test are provided along with 95% confidence intervals (CIs).

School year		Winter break		Spring break		Pseudo‐break	
	ILI?	Before break	After break	Before break	After break	Before break	After break
2014/2015	ILI absences	262	152	74	33	66	88
	Not ILI absences	35,618	32,140	35,806	35,847	35,814	32,204
2015/2016	ILI absences	104	62	275	74	101	131
	Not ILI absences	37,026	37,068	36,855	37,056	37,029	33,286
2016/2017	ILI absences	80	77	115	33	200	108
	Not ILI absences	37,410	33,664	37,375	33,708	37,290	33,633
2017/2018	ILI absences	155	72	69	39	120	71
	Not ILI absences	38,125	34,380	34,383	38,241	34,332	34,381
2018/2019	ILI absences	51	47	224	69	43	116
	Not ILI absences	38,619	38,623	38,446	38,601	27,026	38,554
OR estimate of a‐ILI, after versus before		0.68		0.33		1.00	
OR 95% CI		(0.60, 0.77)		(0.28, 0.38)		(0.87, 1.11)	

### 3.3 Adjusted association between school breaks and a‐ILI

In the regression models, the estimated a‐ILI over the 2‐week period after a break was nearly half the amount of that in the period before a break. The estimated PC following a break was 0.483 (95% CI: 0.347–0.673; *p* < 0.001) for winter break and 0.488 (95% CI: 0.327–0.730; *p* < 0.001) for spring break. The weekly community MAI count was also strongly associated with a‐ILI (*p* ≤ 0.001). No statistical significance was detected in the change in linear or quadratic time components for before versus after breaks (interactions between before/after break and the linear and quadratic time components Table 3). This indicates that the break periods were associated with an overall drop in a‐ILI risk after winter and spring breaks and not a change in the a‐ILI risk over time. In fact, both the linear and quadratic time covariates were non‐significant, indicating that there appears to be no temporal trend in the ±2 weeks around winter and spring breaks.

**TABLE 3:** Summary statistics of the fitted regression model comparing influenza‐like illness (ILI) absenteeism and community medically attended influenza occurring after winter, spring, and pseudo‐breaks to the periods before breaks.

Break type	Coefficient	Estimate	Std. error	z value	*p* value	Estimate 95% CI	Proportional change (PC)	PC 95% CI
Winter	After winter break	−0.73	0.169	−4.29	<0.001	−1.06 — −0.40	0.48	0.35 — 0.67
	Weekly community ILI count	0.03	0.007	8.07	<0.001	0.04 — 0.07	1.06	1.05 — 1.08
Spring	After spring break	−0.72	0.205	−3.50	<0.001	−1.12 — −0.32	0.49	0.33 — 0.73
	Weekly community ILI count	0.05	0.008	5.70	<0.001	0.03 — 0.06	1.05	1.03 — 1.06
Pseudo	After pseudo‐break	0.02	0.165	0.11	0.916	−0.31 — 0.34	1.02	0.74 — 1.41
	Weekly community ILI count	0.01	0.007	1.45	0.146	−0.01 — 0.02	1.01	1.00 — 1.02

**FIGURE 5:**
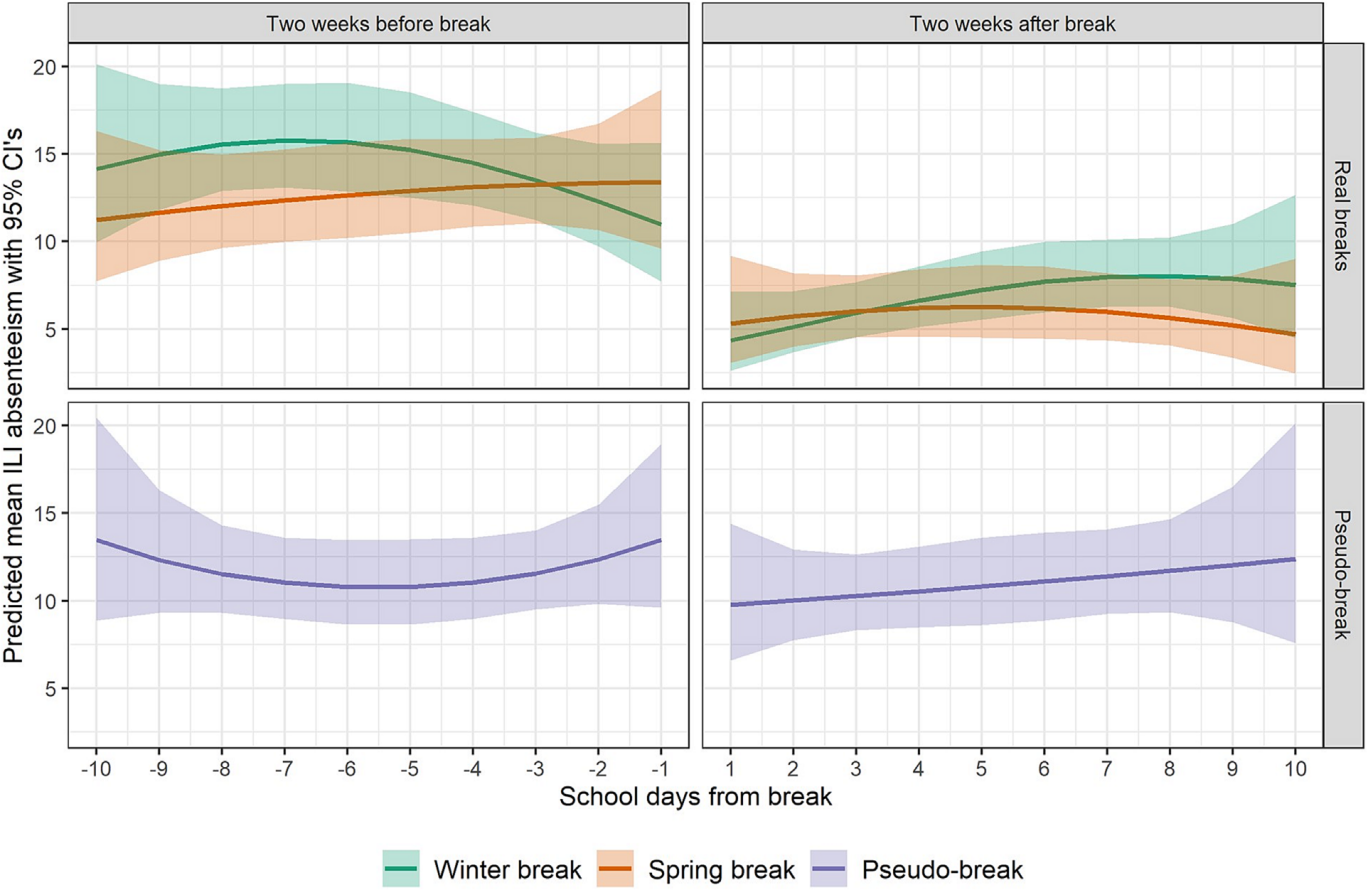
Visualization of break effects: estimated mean absenteeism due to influenza‐like illness (a‐ILI) counts for each of 10 school days before and after winter breaks (green line—upper panel), spring breaks (orange line—upper panel), and pseudo‐breaks (purple line—lower panel) in each of five academic years (please see Table 1 for precise dates and duration of real and pseudo‐breaks). Nine‐day pseudo‐breaks between winter and spring breaks each year were introduced in this analysis to support any findings of true impact from the planned breaks. The 95% confidence intervals are demonstrated by shading. Model estimated an overall reduction in a‐ILI in the periods following break compared with the period before break.

The models produced estimates of daily mean a‐ILI for the 10 days before and after each break are to visually demonstrate the estimated adjusted break effects on a more real‐world scale and are based on the mean weekly community MAI counts and the mean student enrollment of 3749 in OSD (Figure 5). Although the behavior of time remained similar in the 10 days before and after each break, the model consistently estimated an overall reduction in the amount of a‐ILI in the periods following breaks compared with the periods before breaks.

### 3.4 Pseudo‐breaks as a control

There was consistently no statistically significant difference observed in a‐ILI in the 2‐week periods before and after the pseudo‐break when school was actually in session. The unadjusted association between the 2‐week period after the pseudo‐break and the risk for change in a‐ILI estimated an odds ratio of 0.985 (95% CI: 0.872–1.11; *p* = 0.839). The changes in proportions of a‐ILI before and after each pseudo‐break vary throughout the 5 years (Figure 4). All covariates included in the pseudo‐break model were non‐statistically significant (Table 3; full output of all regression models are in Table S1). In Figure 5, the estimated daily ILI means predicted by the model displayed no clear level of change in absenteeism counts for before versus after a pseudo‐break.

## 4 CONCLUSIONS

Over a 5‐year period of enhanced monitoring of cause‐specific absenteeism, from September 2014 to June 2019, a nearly 50% reduction in a‐ILI was observed consistently in the 2‐week periods immediately following scheduled winter and spring breaks with durations of 9–16 days, as compared with the 2 weeks immediately preceding these breaks. We found a strong association between the period indicator and a‐ILI in regression models, in both fully adjusted and unadjusted setups (Table 3; Table S2). We used complex models incorporating non‐linear time, interactions with break, temporal community risk measures, and pseudo‐breaks to adjust for as many break period confounders as was reasonable, thus providing greater confidence that inferences on the effect of before versus after break periods is more likely causal than due to some unmeasured confounder. This implies that the regular scheduled school breaks produce a significant acute effect on a‐ILI. Such an effect has high biological plausibility: (a) if schools are primary centers of influenza transmission and acceleration, and (b) given that the time period spans approximately 2.8–4.4 serial intervals for influenza.^24^

The scale of the proportional differences in a‐ILI associated with each break in Figure 4 appears to reflect the timing of peak influenza circulation and annual seasonal peak across Wisconsin (Figure 2). For example, during the 2014/2015 and 2017/2018 school years, there was relatively widespread circulation before the commencement of winter break, with the seasonal peak occurring in late December and early January.^25^ Thus, winter break appeared to have a larger impact on reducing a‐ILI than spring break in these years. Conversely, in 2015/2016, 2016/2017, and 2018/2019, widespread circulation occurred later in the season with the peak between February and March,^25^ explaining the more profound difference in a‐ILI following spring break. This observation emphasizes the importance of the timing of a school closure on the potential impact on influenza risk.

The absence of significant findings for the pseudo‐breaks lends credence to the true school breaks being an actual causal mechanism to reduce a‐ILI, particularly with the lack of association between pseudo‐breaks and reductions in a‐ILI and weekly community MAI. Although the changes in a‐ILI after the pseudo‐break for any given year in Figure 4 may appear to be significant, the changes are inconsistent with 3 years (2014/2015, 2015/2016, and 2018/2019) having higher a‐ILI following the pseudo‐break and 2 years (2016/2017 and 2017/2018) having lower a‐ILI after the pseudo‐break.

Other results from ORCHARDS—specifically data generated through home visits to a subset of K‐12 students who had to miss school due to an acute respiratory illness—complement the findings from this analysis on school breaks.^20^ Over the five school years (2014–2019), 79% of participants with acute respiratory infections reported missing school because of their illness; 65% of these students who were absent tested positive for influenza or another non‐influenza respiratory viral infection, and more than half thought a classmate or friend was the likely source of infection.^20^ Thus, the ORCHARDS results support the concept that within‐school transmission drives community‐wide outbreaks, and that well‐timed school breaks (or, alternatively, short‐term transitions to distance learning of equivalent duration as a winter or spring break) can reduce influenza or other respiratory virus transmission.

This assessment has several limitations. First, findings based on the models used are suggestive of an association, but do not necessarily imply a causal relationship. These analyses are on observational data, with the main scientific question pertaining to periods occurring before and after the planned breaks, and are by definition ordered through time. Even with non‐linear time effects and a community influenza risk measure in the model, any temporal effects that may impact influenza risk during this time that are unrelated to school breaks would result in confounding between this temporal effects and the period before and after breaks. Second, there is some violation in the assumption of independence of observations in both the adjusted and unadjusted analyses. Because the data used in this assessment were de‐identified and a‐ILI was measured by counts, it is likely that individual students contributed multiple, sequential absences to the a‐ILI counts, thereby altering the independence of daily counts. Third, because parents self‐report absences through the absentee line, there is potential that a‐ILI numbers are underestimated because of underreporting by parents. Fourth, results generated from OSD over five influenza seasons (2014–2019) may not be generalizable to other locations and populations, for markedly different influenza seasons, or over different academic calendars in terms of school break timing relative to local influenza outbreak peaks. Fifth, we used a‐ILI as a proxy for influenza. Whereas we have demonstrated a significant association between influenza virus infection and a‐ILI, we have also shown that influenza type and subtype have differential effects on a‐ILI.^20^ Finally, although community data on MAI were used in an attempt to represent the underlying community risk, the models are imperfect as they do not capture the entirety of the relationship between underlying community level risk and the risk in schools. It is possible that the period indicator is representing differential community‐level risk behaviors during before‐ versus after‐break periods.

Although reports documenting the effect of school closures on reduced influenza transmission exist, there remains a lack of consensus on its effectiveness. The majority of current literature has assessed the impact of reactive school closures during an influenza pandemic.^26^, ^27^, ^28^, ^29^, ^30^, ^31^, ^32^, ^33^ Differences in the timing of implementation and length of closure during the pandemic may explain why studies have found variable results from reactionary closures. Furthermore, there is limited research on the impact of school breaks on absenteeism due to SARS‐CoV‐2 and other respiratory viruses.

Results from these analyses are consistent with findings from other studies looking at the role of scheduled breaks on ILI.^34^, ^35^ A study in South Korea observed an immediate 27%–39% reduction in influenza transmission during the break period, with a 6%–23% reduction in overall transmission following spring break.^34^ Another study found school closures to prevent or delay up to 42% of potential influenza cases among school‐age children.^35^ Although we measured a‐ILI as the outcome in this analysis, previous studies have suggested that observed a‐ILI can adequately represent changes in community influenza.^36^ Moreover, we have previously demonstrated a significant association between a‐ILI and influenza in ORCHARDS.^20^ Furthermore, several studies have proposed that regular school closures may mitigate community impact by changing social mixing patterns.^37^, ^38^, ^39^

Overall, the findings from these analyses support the hypothesis that planned K‐12 school breaks of moderate duration (9–16 days) reduce influenza transmission. Our finding is consistent with the results of the modeling studies which explored the impact of different timing and durations of the school closures during influenza pandemics,^29^ as well as with the conclusions of observational studies of school holidays' effect on influenza transmission in other countries.^12^, ^40^ The identified impact occurs in the short term and does not imply a long‐term effect on an annual seasonal influenza epidemic; however, such short‐term effect may be helpful for targeted suppression of influenza activity to reduce pressures on local health care systems during the local disease surges. Additional analyses investigating the impact of well‐timed shorter breaks, both planned and unplanned, on a‐ILI may determine an optimal duration for brief school closures to effectively suppress community transmission of influenza.

## AUTHOR CONTRIBUTIONS

Cecilia He made a substantial contribution to the conceptualization and design of the article, the acquisition of data, and drafted and revised the article critically. Derek Norton made a substantial contribution to the analysis, interpretation of data for the article, and drafted and revised the article critically. Jonathan Temte made a substantial contribution to the conceptualization and design of the article, acquisition of data, and drafted and revised the article critically. Shari Barlow contributed to the conceptualization and design, and drafted and revised the article critically. Maureen Goss contributed to the conceptualization and design, data curation, and revised the article critically. Emily Temte contributed to the conceptualization and design, data curation, and revised the article critically. Cristalyne Bell contributed to the conceptualization and design, data curation, and revised the article critically. Guanhua Chen contributed to the formal analysis, interpretation of data for the article, and revised the article critically. Amra Uzicanin contributed to the conceptualization and design of the article, and revised the article critically. All authors reviewed the results and approved the final version of the manuscript, and agree to be accountable for all aspects of the work in ensuring that questions related to the accuracy or integrity of any part of the work are appropriately investigated and resolved.

## CONFLICT OF INTEREST STATEMENT

Jonathan L. Temte, MD/PhD reports non‐financial support from Quidel Corporation during the conduct of the study.

### PEER REVIEW

The peer review history for this article is available at https://www.webofscience.com/api/gateway/wos/peer-review/10.1111/irv.13244.

## Supporting information


**Table S1:** Supporting Information.

**Table S2:** Output of unadjusted Poisson regression models.

## Data Availability

The data that support the findings of this study are openly available in Harvard Dataverse at https://doi.org/10.7910/DVN/FCTKNR.
