# Supplementary material for: Effect of planned school breaks on student absenteeism due to influenza‐like illness in school aged children—Oregon School District, Wisconsin September 2014–June 2019
Source: Influenza Other Respir Viruses. 2024 Jan 16;18(1):e13244. doi: 10.1111/irv.13244 (PMC10792089; doi:10.1111/irv.13244)
Supplement: Supplementary file 1 — Table S1: Supporting Information. [file IRV-18-e13244-s001.docx]

**Supplemental Table 1**

| **Break type / model** | **Regression Coefficient** | **Coef. Estimate** | **Std. Error** | **z value** | **p value** | **Estimate 95% CI** | **Proportional change (PC)** | **PC 95% CI** |
| --- | --- | --- | --- | --- | --- | --- | --- | --- |
| Winter | after winter break | -0.73 | 0.17 | -4.29 | <0.001 | -1.06 — -0.40 | 0.48 | 0.35 — 0.67 |
|  | school days from break | 0.03 | 0.03 | 1.11 | 0.265 | -0.02 — 0.08 | 1.03 | 0.98 — 1.08 |
|  | quadratic of school days from break | -0.01 | 0.01 | -1.11 | 0.269 | -0.03 — 0.01 | 0.99 | 0.97 — 1.01 |
|  | weekly community ILI count | 0.06 | 0.01 | 8.07 | <0.001 | 0.04 — 0.07 | 1.06 | 1.05 — 1.08 |
|  | after break x school days | 0.03 | 0.04 | 0.79 | 0.432 | -0.05 — 0.12 | 1.03 | 0.95 — 1.12 |
|  | after break x quadratic school days | 0.00 | 0.02 | -0.16 | 0.875 | -0.04 — 0.03 | 1.00 | 0.97 — 1.03 |
|  |  |  |  |  |  |  |  |  |
| Spring | after spring break | -0.72 | 0.21 | -3.50 | <0.001 | -1.12 — -0.32 | 0.49 | 0.33 — 0.73 |
|  | school days from break | -0.02 | 0.03 | -0.77 | 0.445 | -0.07 — 0.03 | 0.98 | 0.93 — 1.03 |
|  | quadratic of school days from break | 0.00 | 0.01 | -0.22 | 0.823 | -0.02 — 0.02 | 1.00 | 0.98 — 1.02 |
|  | weekly community ILI count | 0.05 | 0.01 | 5.70 | <0.001 | 0.03 — 0.06 | 1.05 | 1.03 — 1.06 |
|  | after break x school days | 0.01 | 0.05 | 0.13 | 0.899 | -0.09 — 0.11 | 1.01 | 0.91 — 1.11 |
|  | after break x quadratic school days | -0.01 | 0.02 | -0.48 | 0.634 | -0.05 — 0.03 | 0.99 | 0.96 — 1.03 |
|  |  |  |  |  |  |  |  |  |
| Pseudo | after fake break | 0.02 | 0.17 | 0.11 | 0.916 | -0.31 — 0.34 | 1.02 | 0.74 — 1.41 |
|  | school days from break | 0.00 | 0.03 | -0.01 | 0.996 | -0.05 — 0.05 | 1.00 | 0.95 — 1.05 |
|  | quadratic of school days from break | 0.01 | 0.01 | 1.05 | 0.295 | -0.01 — 0.03 | 1.01 | 0.99 — 1.03 |
|  | weekly community ILI count | 0.01 | 0.01 | 1.45 | 0.146 | -0.01 — 0.02 | 1.01 | 1.00 — 1.02 |
|  | after break x school days | 0.03 | 0.04 | 0.65 | 0.517 | -0.05 — 0.11 | 1.03 | 0.95 — 1.11 |
|  | after break x quadratic school days | -0.01 | 0.02 | -0.68 | 0.497 | -0.04 — 0.02 | 0.99 | 0.96 — 1.02 |
