# Supplementary material for: Effect of planned school breaks on student absenteeism due to influenza‐like illness in school aged children—Oregon School District, Wisconsin September 2014–June 2019
Source: Influenza Other Respir Viruses. 2024 Jan 16;18(1):e13244. doi: 10.1111/irv.13244 (PMC10792089; doi:10.1111/irv.13244)
Supplement: Supplementary file 2 — Table S2: Output of unadjusted Poisson regression models. [file IRV-18-e13244-s002.docx]

**Supplemental Table 2:** Output of unadjusted Poisson regression models.

| **Break type** | **Coefficient** | **Estimate** | **Std. Error** | **z value** | **p value** | **Estimate 95% CI** | **Proportional change (PC)** | **PC 95% CI** |
| --- | --- | --- | --- | --- | --- | --- | --- | --- |
| Winter | after winter break | -0.40 | 0.15 | -2.64 | 0.008 | -0.70 — -0.10 | 0.67 | 0.50 — 0.90 |
| Spring | after spring break | -1.12 | 0.15 | -7.60 | <0.001 | -1.40 — -0.83 | 0.33 | 0.25 — 0.44 |
| Pseudo | after pseudo break | -0.03 | 0.11 | -0.31 | 0.754 | -0.25 — 0.18 | 0.97 | 0.78 — 1.19 |
